# Arm trajectories and writing strategy in healthy children

**DOI:** 10.1186/1471-2431-12-173

**Published:** 2012-11-07

**Authors:** Matteo Chiappedi, Rosella Togni, Elisabetta De Bernardi, Ilaria Maria Carlotta Baschenis, Sara Battezzato, Umberto Balottin, Elena Dalla Toffola, Maurizio Bejor

**Affiliations:** 1 Don Carlo Gnocchi ONLUS Foundation, Piazzale Morandi 6, Milan, 20121, Italy; 2 Department of Surgical, Resuscitative, Rehabilitative and Transplant Sciences, University of Pavia, Via Aselli 45, Pavia, 27100, Italy; 3 Child Neuropsychiatry Unit, IRCCS “C. Mondino” Foundation, University of Pavia, Via Mondino 2, Pavia, 27100, Italy

## Abstract

**Background:**

Evaluation of elementary writing skills in children is usually obtained with high resolution (and high cost) techniques or with low resolution pen-and-paper tests. In this observational study we tested a quantitative method to obtain normative data to describe arm movement during a writing precursor gesture.

**Methods:**

We recruited 226 healthy children (mean age 9,1 years [range: 6.3 – 11.4 years]), attending primary schools belonging to the “Istituto Comprensivo” of Rivanazzano Terme (Pavia). We asked to drive a cursor through a polygonal path (labyrinth) projected in front of them using a wireless mouse. Dartfish™ video analysis software was used to elaborate images and Excel™, MedCalc™ and Statistica 7™ to analyze values of shoulder, elbow and wrist ranges of motion, arm trajectories, execution times and gesture accuracy.

**Results:**

Differences seen in motor strategies, when divided according to attended class, suggest a proximal-distal maturation of motor control. Obtained values were not significantly correlated with variables such as gender, ethnicity or cognitive functioning.

**Conclusions:**

This type of approach to a study of arm movement during childhood represents a valid alternative to other tests, considering that it can differentiate children who perform similarly in the VMI test and is non-invasive, low-cost and easily reproducible.

## Background

Arm movements are the final result of a complex mechanism, involving motor algorithms structured according to motor experience [1]. The brain network involved is complex and not yet completely understood [2]. Stability and adaptability of motor performances are fundamental to skilled actions throughout the life span [3]. Adults have highly stereotyped reaching movements that lead to stable arm trajectories, with relatively narrow range of motion of arm joints. Studies with complex optoelectronic systems in healthy subjects have shown that upper limb motor trajectories are highly variable before the age of 3 and then tend to became more and more stable; when the child is about 11, adult performance is approached [4]. Moreover, planning an arm gesture implies that the child has the ability to stabilize the head during postural and kinetic activities [5].

Writing can be studied, from a kinetic point of view, as the result of specific periodic movements, inscribed on coordinative patterns, whose control has characteristics similar to a pair of non linear oscillators [6]. This system needs a sufficient integration of visual perception, so that any disorder reducing visual acuity and/or eye coordination can impair it [7]. Weil and Amundson define visual–motor integration as the ability to coordinate visual information with a motor response [8]: an efficient eye-hand coordination is particularly important in pre-school and school years in order to perform writing precursor gestures useful to learn how to write [9].

In a previous work we presented preliminary findings about a new and low-cost assessment system of arm movements [10].

Since optoelectronic devices and other high resolution techniques are too expensive for everyday clinical routine, visuo-motor integration tests such as the VMI [11] are currently used to evaluate motor performance in a pre-writing task. However clinical experience indicates that this approach has limited value in assessing the effect of rehabilitative treatments.

The primary goal of this paper is to present normative data of shoulder, elbow and wrist ranges of motion, execution time and accuracy, in a simple writing precursor gesture. These normative data could be the reference base for future use of the same assessment system in children with movement disorders involving the arm [12]. The second goal of this study is the assessment of factors influencing choices of motor strategies.

## Methods

226 healthy children attending primary school at the Istituto Comprensivo di Rivanazzano Terme (Pavia, Italy) were enrolled for this study (see Table 1 for descriptive statistics). All parents gave informed consent to testing and we strictly followed recommendations from the Helsinki Declaration.

**Table 1:** Description of study subjects

	**I Grade**	**II Grade**	**III Grade**	**IV Grade**	**V Grade**	**Total**
Males	17 (43.6)	25 (54.3)	20 (47.6)	24 (41.4)	21 (51.2)	107 (47.3)
Females	22 (56.4)	21 (45.7)	22 (52.4)	34 (58.6)	20 (48.8)	119 (52.7)
Total	39 (17.3)	46 (20.3)	42 (18.6)	58 (25.7)	41 (18.1)	226 (100)

Data are giving in raw numbers, with percentages in brackets. Totals’ percentages are calculated in relation to total subjects’ number; percentages for males and females in each grade are calculated in relation to subjects attending that grade.

Inclusion criteria were:

• normal pregnancy, birth and neonatal period;

• normal neurologic exam;

• no previous neuropsychiatric consultations;

• no uncorrected eye problem;

• no previous arm injuries.

Our group included no children beginning primary school in advance, nor having to repeat classes. All children were right handed.

All children were assessed using Raven's Colored Progressive Matrices [13] and Visual-Motor Integration test [11].

**Figure 1:**
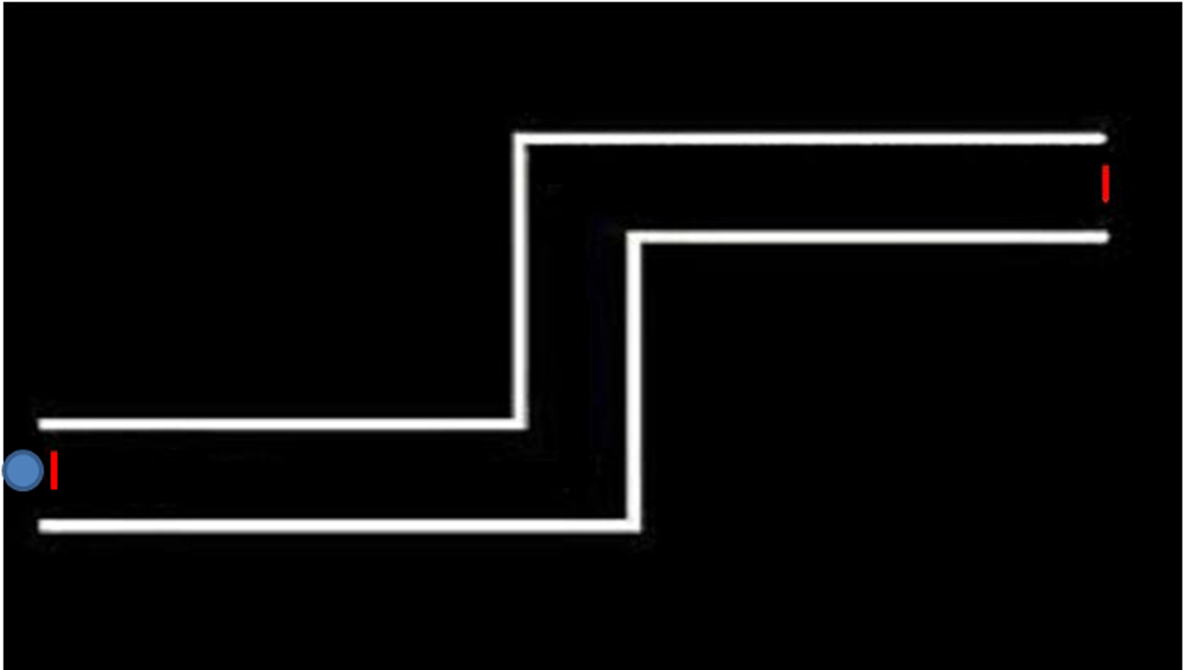
**The maze.** The maze projected in front of the child. The red lines showing the entrance and the exit of the maze were not shown to the child.

**Figure 2:**
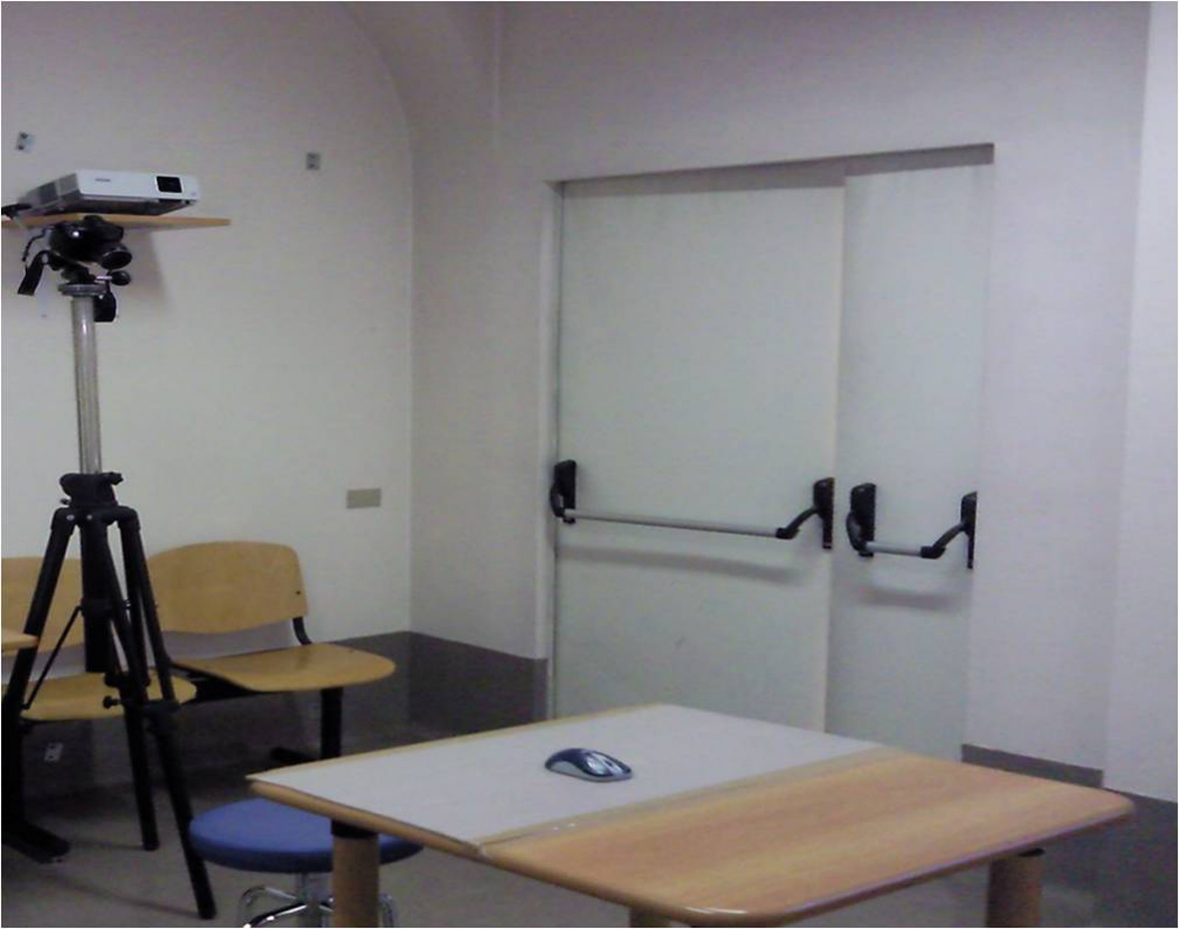
**The setting.** The photograph shows the original setting. As detailed in the text, the camcorder was placed behind the children, 2 meters high and skewed downwards 120 degrees (so to equal humerus inclination on the forearm).

We studied the same gesture used in our preliminary study [10]. It consisted in driving a cursor through a labyrinth projected in front of the child by moving a wireless mouse on a table plane. Orientation was rightwards, to mimic writing. The maze was painted in white, on a black background, and was quite wide (see Figure 1); it was generated with a program we developed and called PRINC – Reaction Times. The sitting position of the subject was adjusted in height using a tripod in order to maintain his back and head straight and the visual perpendicular to the projected labyrinth. In this position the forearm was lying on the table with the elbow flexed at about 120 degrees (see Figure 2).

The child was asked to drive the cursor out of the maze as fast as he could without touching the labyrinth’s walls (FASTER condition) or to try not to “hit” the walls while running the maze (ERROR condition). These different instructions were given in random order to all children, who were therefore assessed twice, with an interval between one session and the other to prevent immediate repetition learning effect.

Their performance was captured with a camcorder CASIO Exilim EX F1, placed behind the child, 2 meters high and skewed downwards 120 degrees (so to equal humerus inclination on the forearm). We assessed shoulder, elbow and wrist angles on the horizontal plane during motor tasks with a sampling rate of 125 Hz, using virtual markers generated by DartFish Pro Suite 5.0™ software and placed on specific bone landmarks.

Statistical analysis was performed using MedCalc 9.5.1™ and Statistica 7™. The following variables were assessed: VMI score, RPM score, joint ranges of motion, time taken to complete each task and number of mistakes. In the whole sample all variables were normally distributed (Kolgomorov-Smirnov test; P<0.01 or more significant), therefore Pearson’s correlation coefficient was used to assess correlations between the variables measured by our system and the scores obtained in the VMI and RPM tests.

We also performed a multivariate test of significance by means of a MANOVA design assuming that age (attended class; primary school in Italy is divided in 5 grades and we had excluded children having repeated any class) or sex could explain differences in mean values obtained for joint ranges of motion. Specific comparisons between different grades (one grade versus the next one) were conducted to identify the age of changes in motor strategy; uncorrected post-hoc Student’s t was used, considering P=0.01 or lower as significant to balance the risk of Type I and Type II errors.

## Results

Children’s performances in neuropsychological assessment are shown in Table 2. As expected, most children had a normal or even above average performance in the cognitive test (RPM): this was probably due to the exclusion of children with known neuropsychiatric disorders. VMI results were distributed approximately as expected in a non clinical sample.

**Table 2:** Neuropsychological findings in study subjects

**Class**	**VMI (%)**	**RPM (%)**
0	8 (3.5)	7 (3.1)
1	71 (31.4)	16 (7.1)
2	146 (64.6)	129 (57.1)
3	1 (0.5)	44 (19.4)
4	0 (0)	30 (13.3)

VMI score in classified according to test manual as: 0 very low; 1 low; 2 average; 3 good; 4 very good. CPM score is classified as: 0 very low (<5th percentile), 1 borderline (between 5th and 25th percentile), 2 normal (between 25th and 75th percentile), 3 good (between 75th and 95th percentile), 4 very good (over 95th percentile).

There was no significant correlation between the RPM and VMI scores and the variables measured with our assessment technique (joint ranges of motion, time to complete tasks, number of errors made; r values were between – 0.1 and + 0.230; all p values were over 0.4).

According to our MANOVA model, in the overall analysis attended grade (F=1.615; p=0.005) but not sex (F=1.726; p=0.059) correlated with measured joint ranges of motion.

Range of motion of wrist, elbow and shoulder under both test conditions (FASTER and ERROR) are shown in Table 3.

**Table 3:** Joint ranges of motion wrist, elbow and shoulder range of motion (in degrees)

		**ERROR condition**		**FASTER condition**	
Class	Joint	Mean	S.D.	Mean	S.D.
I	Wrist	9.69	5.60	10.23	6.02
	Elbow	10.01	4.42	13.35	10.94
	Shoulder	6.43	4.47	6.64	3.41
II	Wrist	9.50	0.95	9.77	5.23
	Elbow	9.01	3.90	9.66	5.07
	Shoulder	4.16	2.78	4.07	2.29
III	Wrist	10.67	5.43	10.53	5.30
	Elbow	7.12	3.26	7.72	3.74
	Shoulder	3.43	1.77	4.31	2.37
IV	Wrist	10.09	5.11	10.86	4.86
	Elbow	9.28	5.12	11.54	10.05
	Shoulder	4.00	2.61	4.95	3.13
V	Wrist	12.18	4.58	11.57	6.03
	Elbow	8.19	4.40	9.17	6.39
	Shoulder	3.42	1.81	4.39	2.79

Under ERROR condition, shoulder range of motion was significantly higher in children attending first grade of primary school (p=0.006 compared to those attending second class). Elbow range of motion showed a statistically significant change between children attending second and third grade (p=0.008). Significant differences in wrist range of motion were found between children attending fourth and fifth grade (p=0.01).

Under FASTER condition, shoulder range of motion was again significantly higher in children attending first grade than in those attending second grade (p=0.0003). The same applied to elbow range of motion (p=0.005). There were no significant differences in wrist range of motion.

As expected, under ERROR condition children made a lower number of errors (i.e. touched the walls of the labyrinth less often); time used to complete each task instead was not significantly different.

These data were then plotted in a Cartesian plane, comparing angle variations (y) to time (x); all the curve of fitting calculated in this way were second order equation and could be expressed as y = ax^2^ + bx + c (as expected from literature findings [14]). The average values were used to plot six functions, each representing the angle of a given joint (shoulder, elbow or wrist) according to time under one of the two conditions (FASTER or ERROR).

After checking that fitting coefficients (the “a” in the above reported equation) were normally distributed (Kolgomorov-Smirnov test; p>0.05), we also plotted the −2, -1 and +1, +2 standard deviation’s curves for each joint and condition.

**Figure 3:**
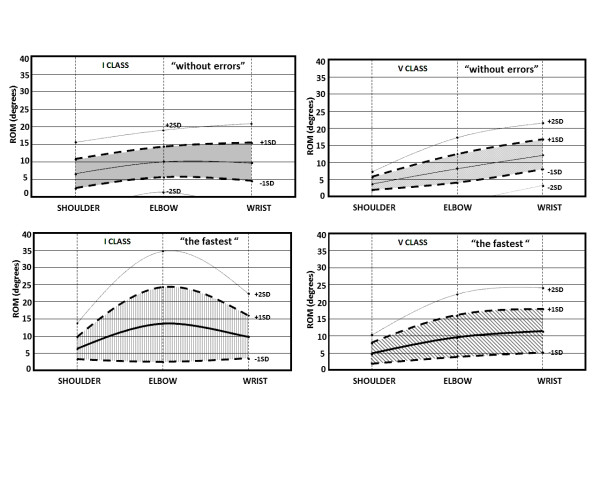
**Representation on the cartesian plane of upper limb trajectories.** These trajectories are described by parabolic curves that corresponds to mean, -1;-2 and +1;+2 standard deviation curves. First line: shoulder, elbow and wrist angles of children attending first and fifth class under ERROR condition. Second line: shoulder, elbow and wrist angles of children attending first and fifth class under FASTER condition. Blue areas account for 80% of children in each group.

Figure 3 shows the results we obtained. The first line shows shoulder, elbow and wrist angles of children attending first and fifth class under ERROR condition, while the second line shows the same parameters under FASTER condition. Values are given in detail in Table 3.

We found no significant differences in neuropsychological tests or in any parameter of our assessment comparing boys and girls.

## Discussion

The first goal of our study was to determine normative data in a non clinical population of school-aged children. Children were quite attracted by our test, because it looked almost like a common videogame to them.

Our assessment method proved able to discriminate the different motor strategies applied in all groups of children. As expected the motor strategy partially changed according to child’s age and task condition. Under ERROR condition, the shoulder showed the widest range of motion in younger children (first grade), while the elbow had the widest range of motion in third grade children and the wrist among older children (fifth grade). These changes in the motor strategy determined an increase in accuracy (i.e. children made less errors), but had no statistically significant effect on the time needed to complete the task.

This proximal-distal age-dependent maturation of motor control is in line with already published findings. Hay [15] showed that children between 4 and 6 years of age can make reaching movements without visual feedback with reasonable accuracy; then, at the age of 7, there is an abrupt reduction of accuracy in reaching ability. The accuracy of reaching ability then begins to increase again and adult performance is approached by 10–11 years of age. Thus, the age of 7 is a transition time in the development of reaching [16].

Under FASTER condition, all children used mainly movements of the elbow to complete the task, except for the younger (first grade) that used the same motor strategy described under ERROR condition (i.e. they used mainly shoulder movements). Nevertheless and as expected [17], younger children showed slower, more variable, less smooth and less linear arm movements than older ones (Figure 2). This observation may imply that age is a relevant factor for accuracy and not for quickness of simple gestures and/or that neural networks controlling accuracy and quickness follow different time schedules. It is also possible that a nurture factor is involved, considering that the first two years of primary school are seen as the time for maturation and reduction of inter-subject variability, so that the diagnosis of Specific Writing Disorder can be formulated only after that period [18]. This is also important because none of our children had symptoms or neurological signs of Developmental Coordination Disorder [19].

Many studies have addressed the question of whether or not motor performance, in terms of speed, accuracy or both, differ between the sexes at any stage of development [20,21]. Sex-related differences in motor task performance and in motor learning are reported in children and they seem to increase after puberty [22]. The lack of statistically significant differences between boys and girls in our study seems however in line with recent hypotheses suggesting that sociological factors (such as reduction of motor activities or increase in console use in both male and female children) could be narrowing the previously described differences [21].

## Conclusions

We believe that our test could be useful, since it provides a valid alternative to other tools. It can differentiate children who perform similarly in the VMI test and is non-invasive, low-cost and easily reproducible. As for any evaluation tool, including neuropsychological tests [23], results obtained need to be understood in the context of the global evaluation of the child. However the possibility to obtain quantitative data that describe arm movement could be useful for assessment and follow up of children with motor difficulties.

A further step in our work will be to use this assessment procedure in the evaluation of children receiving rehabilitative treatment for specific or non-specific delays in writing skills. The normative data we have defined with this study could be used as a reference for interpreting changes due to rehabilitation, using our assessment tool alone or compared to other currently used tests [24]. This could lead to a better tailored rehabilitation, with stronger evidences of efficacy, in a way similar to what has been done for disturbances following brain damage [25]. This quantitative evidence of efficacy could in turn increase parental satisfaction and compliance with the treatment proposed [26].

## Competing interests

None of the authors has competing interests to disclose.

## Authors' contributions

MC, RT, EDB, IMCB, SB performed patients’ testing. MC, UB, EDT and MB conceived of the study, and participated in its design and coordination and helped to draft the manuscript. MC and MB performed the statistical analysis. All authors read and approved the final manuscript.

## Pre-publication history

The pre-publication history for this paper can be accessed here:

http://www.biomedcentral.com/1471-2431/12/173/prepub

## References

[B1] Wolpert DM Gharahmani Z Computational Principles of Movement Neuroscience Nat Neurosci 2000 3 suppl 1212 1217 11127840 10.1038/81497

[B2] D'Angelo E Neuronal circuit function and dysfunction in the cerebellum: from neurons to integrated control Funct Neurol 2010 25 3 125 127 21232207

[B3] Schneiberg S Sveistrup H McFadyen B McKinley P Levin MF The Development Of Coordination For Reach-To-Grasp Movements In Children Exp Brain Res 2002 146 2 142 154 10.1007/s00221-002-1156-z 12195516

[B4] Sveistrup H Schneiberg S McKinley PA McFadyen BJ Levin MF Head, arm and trunk coordination during reaching in children Exp Brain Res 2008 188 2 237 247 10.1007/s00221-008-1357-1 18392615

[B5] Assaiante C Mallau S Viel S Jover M Schmitz C Development of postural control in healthy children: a functional approach Neural Plast 2005 12 2–3 109 118 16097479 10.1155/NP.2005.109 PMC2565455

[B6] Athenes S Sallago I Zanone PG Albaret JM Evaluating The Coordination Dynamics Of Handwriting Hum Mov Sci 2004 23 621 641 10.1016/j.humov.2004.10.004 15589625

[B7] Racine MB Majnemer A Shevell M Snider L Handwriting Performance In Children With Attention Deficit Hyperactivity Disorder (ADHD) J Child Neurol 2008 23 4 399 406 10.1177/0883073807309244 18401033

[B8] Weil MJ Amundson SJ Relationship between visuomotor and handwriting skills of children in kindergarten Am J Occup Ther 1994 48 11 982 988 10.5014/ajot.48.11.982 7840134

[B9] Van Hoorn JF Maathuis CGB Peters LHJ Hadders-Algra M Handwriting, visuomotor integration, and neurological condition at school age Dev Med Child Neurol 2010 52 941 947 10.1111/j.1469-8749.2010.03715.x 20561005

[B10] Chiappedi M De Bernardi E Dalla Toffola E Bejor M Child visuomotor skills: preliminary findings using a new low-cost movement analysis method Funct Neurol 2010 25 1 45 48 20626996

[B11] Preda C Developmental Test of Visual-Motor Integration 2000 Italian Firenze: Giunti OS

[B12] Negrini S Evidence (and research) are the only possible basis of medicine Eur J Phys Rehabil Med 2011 47 189 191 21597432 10.1016/j.rh.2011.05.008

[B13] Belacchi C Scalisi TG Cannoni E Cornoldi C Coloured Progressive Matrices 2008 Italian Firenze: Giunti OS

[B14] Polyakov F Drori R Ben-Shaul Y Abeles M Flash T A compact representation of drawing movements with sequences of parabolic primitives PLoS Comput Biol 2009 5 7 e1000427 10.1371/journal.pcbi.1000427 19578429 PMC2699652

[B15] Hay L Accuracy of children on an open-loop pointing task Percept Mot Skills 1978 47 3 Pt 2 1079 1082 745878 10.2466/pms.1978.47.3f.1079

[B16] Van Dellen T Kalverboer AF Single movement control and information processing, a developmental study Behav Brain Res 1984 12 2 237 239 10.1016/0166-4328(84)90115-3

[B17] Yan JH Thomas JR Stelmach GE Thomas KT Developmental features of rapid aiming arm movements across the lifespan J Mot Behav 2000 32 2 121 140 10.1080/00222890009601365 11005944

[B18] Società Italiana di NeuroPsichiatria Infantile (SINPIA) Linee guida per i disturbi di apprendimento – Parte I: I disturbi specifici di apprendimentowww.sinpia.eu/atom/allegato/146.pdf

[B19] Chang SH Nan-Ying Y Characterization of motor control in handwriting difficulties in children with or withour developmental coordination disorder Dev Med Child Neurol 2009 52 244 250 20002122 10.1111/j.1469-8749.2009.03478.x

[B20] De Bellis MD Keshavan MS Beers SR Hall J Frustaci K Masalehdan A Noll J Boring AM Sex differences in brain maturation during childhood and adolescence Cereb Cortex 2001 11 6 552 557 10.1093/cercor/11.6.552 11375916

[B21] Ghezzo A Guerini FR Bolognesi E Matteoli M Manca S Sotgiu S Bejor M Clerici M Chiappedi M Neuropsycological gender differences in healthy individuals and in pediatric neurodevelopmental disorders. A role for SNAP-25 Med Hypotheses 2009 73 6 978 980 10.1016/j.mehy.2009.05.045 19713048

[B22] Dorfberger S Adi-Japha E Karni A Sex differences in motor performance and motor learning in children and adolescents: an increasing male advantage in motor learning and consolidation phase gains Behav Brain Res 2009 198 1 165 171 10.1016/j.bbr.2008.10.033 19026692

[B23] Chiappedi M Baschenis IMC Dolci R Bejor M Importance of a critical reading of neuropsychological testing Minerva Pediatr 2011 63 3 239 245 21654605

[B24] Feder KP Majnemer A Handwriting development, competency, and intervention Dev Med Child Neurol 2007 49 312 317 10.1111/j.1469-8749.2007.00312.x 17376144

[B25] Zoccolotti P Cantagalli A De Luca M Guariglia C Serino A Trojano L Selective and integrated rehabilitation programs for disturbances of visual/spatial attention and executive function after brain damage: a neuropsychological evidence-based review Eur J Phys Rehabil Med 2011 47 123 147 21448124

[B26] Chiappedi M Maltagliati S Amoruso A Dolci R Carniglia C Bejor M Child rehabilitation refusal: why it happens and possible strategies to avoid it Eur J Phys Rehabil Med 2009 45 4 487 492 20032906

